# Primary Hyperparathyroidism: The Influence of Bone Marrow Adipose Tissue on Bone Loss and of Osteocalcin on Insulin Resistance

**DOI:** 10.6061/clinics/2016(08)09

**Published:** 2016-08

**Authors:** Maira L. Mendonça, Sérgio L. Batista, Marcello H. Nogueira-Barbosa, Carlos E.G. Salmon, Francisco J.A. de Paula

**Affiliations:** I Universidade de São Paulo, Faculdade de Medicina de Ribeirão Preto, Departamento de Medicina Interna, Ribeirao Preto/SP, Brazil; II Universidade de São Paulo, Faculdade de Filosofia, Ciências e Letras de Ribeirão Preto, Departamento de Física, Ribeirão Preto/SP, Brazil

**Keywords:** Primary Hyperparathyroidism, Osteoporosis, Bone Marrow Adipose Tissue, Insulin, Osteocalcin

## Abstract

**OBJECTIVES:**

Bone marrow adipose tissue has been associated with low bone mineral density. However, no data exist regarding marrow adipose tissue in primary hyperparathyroidism, a disorder associated with bone loss in conditions of high bone turnover. The objective of the present study was to investigate the relationship between marrow adipose tissue, bone mass and parathyroid hormone. The influence of osteocalcin on the homeostasis model assessment of insulin resistance was also evaluated.

**METHODS:**

This was a cross-sectional study conducted at a university hospital, involving 18 patients with primary hyperparathyroidism (PHPT) and 21 controls (CG). Bone mass was assessed by dual-energy x-ray absorptiometry and marrow adipose tissue was assessed by ^1^H magnetic resonance spectroscopy. The biochemical evaluation included the determination of parathyroid hormone, osteocalcin, glucose and insulin levels.

**RESULTS:**

A negative association was found between the bone mass at the 1/3 radius and parathyroid hormone levels (r = -0.69; *p*<0.01). Marrow adipose tissue was not significantly increased in patients (CG = 32.8±11.2% *vs* PHPT = 38.6±12%). The serum levels of osteocalcin were higher in patients (CG = 8.6±3.6 ng/mL *vs* PHPT = 36.5±38.4 ng/mL; *p*<0.005), but no associations were observed between osteocalcin and insulin or between insulin and both marrow adipose tissue and bone mass.

**CONCLUSION:**

These results suggest that the increment of adipogenesis in the bone marrow microenvironment under conditions of high bone turnover due to primary hyperparathyroidism is limited. Despite the increased serum levels of osteocalcin due to primary hyperparathyroidism, these patients tend to have impaired insulin sensitivity.

## INTRODUCTION

Parathyroid hormone (PTH) is a crucial element in the complex process of bone development and maintenance. Constantly high 1 or low 2 serum levels of PTH have detrimental effects on bone. Conversely, intermittent exposure of bone cells to high circulating levels of PTH causes bone remodeling to become an anabolic response 3.

While improvements in imaging technology allow visualization of even mild states of primary hyperparathyroidism (PHPT) that systemically affect the skeleton, a gap exists in the knowledge of the mechanism of bone loss in PHPT. Recently, concomitant increases in bone marrow adiposity in several conditions have been shown to be associated with a negative bone balance 4, such as secondary osteoporosis due to anorexia nervosa 5. These disorders are associated with low bone turnover and decreased osteoblast activity, whereas in PHPT, bone loss typically occurs within a microenvironment of unrestrained osteoblast function 6. Because osteoblasts and adipocytes originate from the same mesenchymal stem cells, knowledge of marrow composition in states of high bone turnover is crucial for understanding, at least in part, the mechanism of bone loss in PHPT.

Osteocalcin (OC) produced by osteoblasts is suggested to be the bone link in a new axis involving bone and β-cell secretion of insulin, as well as insulin sensitivity 7,8. While insulin directly stimulates osteoblast activity, OC regulates insulin secretion and action. Evidence of insulin resistance 9 and an increased incidence of diabetes mellitus have been found in PHTP patients 10. Impaired insulin action has been attributed to high calcium levels and hypophosphatemia in previous studies 11. However, serum levels of OC are elevated in PHPT patients and decrease after successful parathyroidectomy 12.

Therefore, the primary objective of the present study was to measure bone marrow adipose tissue (BMAT) in patients with PHPT. Because the association of serum OC levels with insulin resistance has been scarcely evaluated in PHPT patients, the second objective of the present study was to investigate the relationship between serum levels of OC and both insulin and the homeostasis model assessment of insulin resistance (HOMA-IR).

## MATERIALS AND METHODS

### Subjects

The study was conducted on two groups, including control (CG, n = 21) and PHPT patients (PHPTG, n = 18), who were matched for gender, age, weight, height and body mass index. All patients were followed-up at the outpatient clinic of Osteometabolic Disorders of the University Hospital, Ribeirão Preto Medical School, Universidade de São Paulo. The exclusion criteria for both groups were as follows: alcohol abuse, smoking, hepatic or renal disease, or using medications known to interfere with mineral metabolism (e.g., anabolic steroids, glucocorticoids, anticonvulsants, diuretics or medications for the treatment of osteoporosis). The Research Ethics Committee of the University Hospital approved the study (protocol #13898/2010) and all participants gave written informed consent.

### Methods

A blood sample was obtained under standard conditions between 8 and 9 am after an overnight fast. Serum aliquots were used for the determination of biochemical, hormonal and bone-related parameters. The biochemical parameters [albumin, alkaline phosphatase (ALP), calcium, creatinine, ionized calcium, glucose and phosphorus levels] were immediately measured with an autoanalyzer (CT 600i, Wiener Lab, Buenos Aires, Argentina). Individual aliquots for the determination of insulin, iPTH, 25-hidroxy vitamin D (25-OHD) and OC were frozen at -70^o^ C until measurements were obtained.

Insulin was measured by an immunoradiometric assay (Insulin IRMA – Beckman Coulter, Immunotech, Prague, Czech Republic). The Immulite I system (Siemens, Los Angeles, CA, USA) was used to determine iPTH levels, while bone-specific alkaline phosphatase (BAP) and 25-OHD levels were determined by chemiluminescence (Liaison, Diasorin, Saluggia, VC, Italy). OC levels were determined by an immunoenzymometric assay (hOst ELISA – Diasource, Louvain la Neuve, Belgium).

Dual energy X-ray absorptiometry (DXA) and ^1^H magnetic resonance spectroscopy (MRS)

The bone mineral density (BMD) of the lumbar spine (L1-L4), femoral neck, total hip and 1/3 radius were determined by DXA (Discovery, Hologic Inc., Bedford, MA, USA). The precision errors were 1.2% for L1-L4, 2.3% for the femoral neck, 2.7% for the total hip and 1.7% for the 1/3 radius.

The fat fraction (FF) was estimated by proton magnetic resonance spectroscopy (^1^H-MRS) using a 1.5 Tesla scanner, as previously described 13. In brief, a sagittal spin echo sequence (TR/TE = 3900/120 ms) was acquired to localize the L3 vertebral body and to identify pre-existing abnormalities of the lumbar vertebrae. A voxel measuring 20×20×20 mm^3^ was placed within the L3 vertebral body. Single-voxel ^1^H-MRS data were acquired using a PRESS sequence without water suppression with the following parameters: TR of 1600 ms, 16 averages, 2048 data points, and a spectral bandwidth of 1500 Hz. The spectra were acquired with several echo times, i.e., TE = 25, 35, 50, 60 and 80 ms, to consider the relaxation effect on the water and lipid content estimation. The spectra were processed with the LC-model software (http://s-provencher.com/pages/lcmodel.shtml). A mean lipid peak of 1.3 ppm was used for fat quantification. The FF was estimated as the lipid-lipid plus water ratio without the relaxation effect.

### Statistical analysis

Student’s t-test was used to compare the results obtained for the two groups. The Pearson correlation test was used to evaluate the relationship between two parameters. Given the skewed distribution of iPTH and OC levels, a base-10 logarithm transformation was used for the analysis. The analysis was performed using R software version 2.15.3 (http://www.R-project.org). The results are reported as the means ± standard deviations, with the level of significance set at *p* < 0.05.

## RESULTS

Table 1 shows the clinical characteristics and results of the laboratory tests for both groups. Serum calcium, total alkaline phosphatase and iPTH levels were significantly increased in the PHPTG, while the circulating levels of inorganic phosphorus and 25-OHD were higher in the CG. Sixteen PHPTG subjects (88.9%) had osteoporosis as determined by the BMD T-Score. Table 1 shows that the serum levels of OC were approximately 4 times higher in the PHPTG than in the CG (*p* < 0.005).

**Figure 1:**
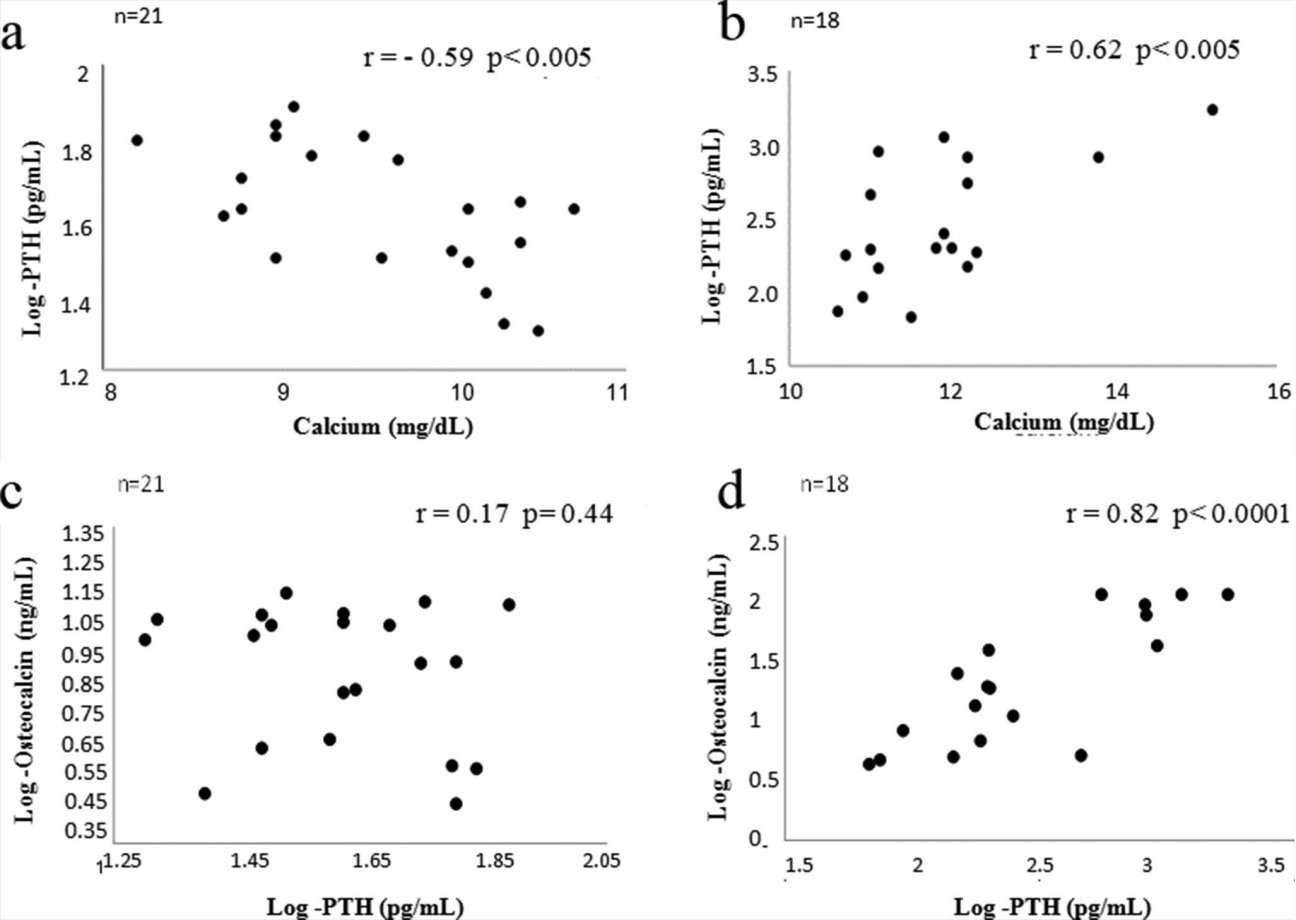
Correlation between the iPTH serum levels (log iPTH) and the calcium serum levels in the control group (a) and in patients with primary hyperparathyroidism (b). Correlation between the iPTH (log iPTH) serum levels and the osteocalcin (log osteocalcin) serum levels in the control group (c) and in patients with primary hyperparathyroidism (d).

The CG showed a negative correlation between serum calcium and iPTH levels (*p* < 0.005, r = -0.59; CI = -0.81 to -0.20) (Figure 1a). Conversely, PHPTG subjects exhibited a positive association of iPTH with calcium levels (*p* < 0.01, r = 0.61; CI = 0.36−0.88) (Figure 1b) and OC (*p* < 0.0001; r = 0.82; CI = 0.59 to 0.93) (Figure 1d). No relationship was observed between the serum levels of iPTH and 25-OHD.

PHPT patients exhibited lower a BMD of the 1/3 radius than CG subjects (CG = 0.676 ± 0.074 *vs* PHTPG = 0.578 ± 0.132 g/cm^2^, *p* < 0.01; CI = -0.17 to -0.03). No significant difference was found in bone mass between the two groups in the lumbar spine (CG = 0.958 ± 0.106 *vs* PHPTG = 0.879 ± 0.194 g/cm^2^), femoral neck (CG = 0.775 ± 0.125 *vs* PHPTG = 0.752 ± 0.190 g/cm^2^) or total hip (CG = 0.917 ± 0.090 *vs* PHPTG = 0.862 ± 0.180 g/cm^2^). Negative correlations of iPTH levels with the BMD of the 1/3 radius (r = -0.69; *p* < 0.005; CI = -0.87 to -0.3348), lumbar spine (r = -0.483; *p* < 0.05, CI = -0.77 to -0.02), femoral neck (r = -0.537; *p* < 0.05; CI = -0.80 to -0.09) and total hip (*p* < 0.05; r = -0.57; CI = -0.82 to -0.14) were observed only in the PHPTG. The BMD was not associated with serum levels of 25-OHD in either group.

**Figure 2:**
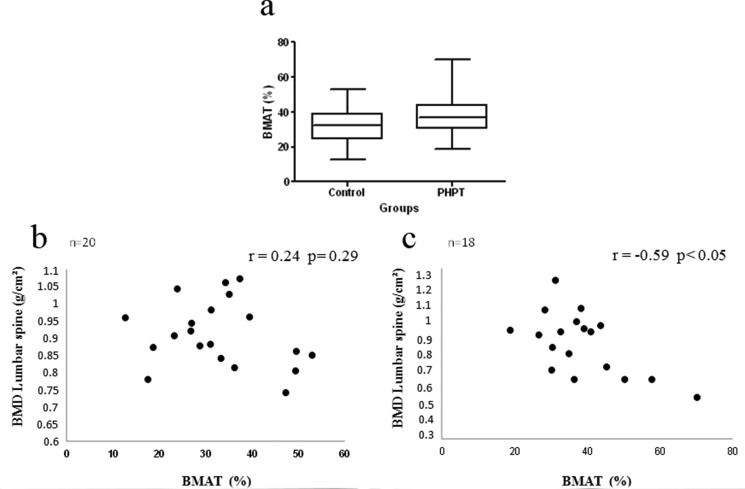
Bone marrow adipose tissue in the control group and in patients with primary hyperparathyroidism (a). Correlation between bone marrow adipose tissue (BMAT) and the bone mineral density of the lumbar spine in the control group (b) and in patients with primary hyperparathyroidism (c).

The BMAT did not differ significantly between groups (CG = 32.8 ± 11.2% *vs* PHPTG = 38.6 ± 12.4%) (Figure 2a). A negative correlation was observed between lumbar spine BMD and BMAT only in the PHPTG (*p* < 0.05; r = -0.59; CI = -0.75 to 0.02) (Figure 2b and 2c). While a negative correlation was observed between BMAT values and serum levels of PTH in the CG, no relationship between these parameters was observed in the PHPTG. The BMAT was not associated with serum levels of 25-OHD or insulin or HOMA-IR values in the two groups.

Serum glucose levels were similar in the two groups. However, the PHPTG tended to have higher serum insulin levels (*p* = 0.059) and HOMA-IR values (*p* = 0.06) than the control group. No correlations were observed for serum levels of insulin, glucose and HOMA-IR values with serum levels of OC.

## DISCUSSION

Recently, new findings in animal models have shown that bone and mineral metabolism are intimately linked to energy metabolism. For instance, the total or partial deletion of vitamin D receptors in mice leads to bone loss but also produces a lean phenotype and metabolic advantages 14,15. Undercarboxylated OC (unOC) has been shown to improve glucose tolerance and insulin resistance in mice with insulin receptor deletion specifically in osteoblasts 7. Finally, bone marrow contains a unique type of adipose tissue with an unknown function. It has been hypothesized that BMAT may provide energy support for bone cells during bone development, but it can also have detrimental effects in several conditions, such as aging, hypercortisolism and food deprivation 4. The present study demonstrated that PHPT patients do not show significantly increased BMAT but exhibit a negative relationship between BMAT and BMD in the lumbar spine. In addition, despite their increased serum OC levels, PHPT patients show a concomitant tendency toward high serum insulin levels and HOMA-IR values. In other words, high serum OC levels are not associated with improved insulin sensitivity in PHPT.

Consistent with previous studies, an evaluation based on DXA showed that PHPT has a greater effect on cortical bone than trabecular bone 16,17. Bone mass was significantly lower in PHPT patients than in controls only at the 1/3 radius, while a non-significant difference was observed at the other sites. It should be emphasized that this point has been recently considered important because improvements in imaging technology have shown that trabecular bone is not spared in PHPT 18. In support of this new evidence, the present study showed a negative relationship of serum PTH levels not only with the BMD of the 1/3 radius but also with the lumbar spine BMD, a bone site predominantly composed of trabecular bone.

BMAT was not significantly increased in PHPT patients, but a negative correlation was found between BMAT and BMD at the lumbar spine. Previous studies investigating the content of BMAT in two conditions causing secondary osteoporosis (i.e., anorexia nervosa and hypercortisolism) reported a significantly increased amount of BMAT. In contrast to PHPT, anorexia nervosa and glucocorticoid-induced osteoporosis result in low osteoblastic activity. Most likely, these different results reflect the diverse mechanisms of bone loss that depend on the circumstances. *In vitro* studies that have investigated the catabolic effect of PTH have suggested that PTH-induced osteoclast activity requires the presence of osteoblastic cells 19. Moreover, previous studies have also shown that PTH enhances the commitment of progenitor cells to differentiate into osteoblasts 20. Therefore, it can be hypothesized that the imbalance of osteogenesis and adipogenesis is less accentuated in PHPT than in anorexia nervosa and hypercortisolism.

Recently, two independent groups have used animal models to show that OC, particularly its unOC form, is a potential systemic modulator of glucose metabolism 7,8. However, contradictory results have been observed in clinical investigations regarding the role of total OC and unOC in glucose metabolism. Pittas et al. observed that OC is negatively associated with fasting circulatory levels of insulin and glucose in individuals older than 65 years 21. Conversely, antiresorptive drugs, such as alendronate 22, which indirectly reduce OC levels, do not impair glucose tolerance. Consistent with the latter study, our data did not show an association between the HOMA-IR index and the total OC level or a relationship between OC and glucose or insulin. Moreover, previous studies have indicated that hypercalcemia 23 and hypophosphatemia 11 could be directly involved with the emergence of hyperinsulinemia in PHPT patients. Therefore, the role of OC in the circulatory levels of insulin as a causative or concomitant factor in primary hyperparathyroidism still needs to be determined. Taken together, our results show that despite their high serum levels of OC, PHPT patients have no improvement in insulin sensitivity. In addition, the present study reinforces recent data showing no relationship between BMAT and both serum insulin levels and HOMA-IR values 13.

The present study shows that bone marrow adipose tissue is not significantly increased in patients with PHPT. These results suggest that bone loss in PHPT occurs at a lower imbalance between osteoblastogenesis and adipogenesis in the bone marrow microenvironment than in anorexia nervosa. Although iPTH is strongly correlated with serum OC levels, no relationship exists between serum OC levels and both serum glucose levels and HOMA-IR values. In addition, no correlation exists between BMAT and insulin resistance.

## AUTHOR CONTRIBUTIONS

De Paula FJ was responsible for the research proposal, designed the study, discussed all the results and wrote the manuscript. Mendonca ML participated in the study design, volunteer selection, data collection, results analysis and manuscript preparation. Batista SL participated in the design of the study, was directly involved in the assessment of BMD and contributed to the preparation of the manuscript. Nogueira-Barbosa MH and Salmon CE participated in the design of the study, were directly involved in the assessment of bone marrow adipose tissue and contributed to the preparation of the manuscript.

## Figures and Tables

**Table 1:** Clinical characteristics and biochemical evaluation of control subjects (CG) and patients with primary hyperparathyroidism (PHPTG).

	CG (n = 21)	PHPTG (n = 18)	Estimated difference [C.I. (95%)]	*p*-value
Age (years)	50.1±9.4	51.1±11.3	-0.96 (-7.66;5.74)	0.77
Weight (kg)	73.3±13.6	76.5±21.6	-3.2 (-14.81;8.29)	0.57
Height (m)	1.65±0.1	1.61±0.1	0.04 (-0.01;0.10)	0.14
BMI (kg/m^2^)	26.8±4.1	29.8±6.8	-2.9 (-6.52;0.64)	0.10
Total calcium (mg/dL)	9.6±0.7*	11.9±1.1	-2.3 (-2.88;-1.66)	*<*0.01
Albumin (g/dL)	4.4±0.3	4.2±0.3	0.1 (-0.36;0.04)	0.11
Albumin corrected calcium (mg/dL)	9.31±0.76*	11.6±1.3	-2.41 (1.76;3.06)	*<*0.01
Phosphorus (mg/dL)	3.4±0.4*	2.6±0.6	0.8 (0.48;1.15)	<0.01
Alkaline phosphatase (U/L)	174.2±49.4*	364.4±252.0	-190.2 (-303.9;-76.42)	*<*0.005
Creatinine (mg/dL)	0.8±0.2*	1.1±0.4	-0.3 (-0.06;0.46)	<0.05
iPTH (pg/mL)	44.5±16.7	459.8±462.2	-413.3 (-641.8;-184.9;)	<0.005
Log_10_ iPTH (pg/mL)	1.62±0.17*	2.46±0.42	-0.85 (-1.049; -0.66)	*<*0.01
25-hydroxyvitamin D (ng/mL)	24.7±3.9*	18.3±4.8	6.4 (3.6;9.2)	<0.01
Osteocalcin (ng/mL)	8.65±3.6*	36.5±38.4	-27.03 (-45.66;-8.39)	*<*0.05
Log_10_ Osteocalcin (ng/mL)	0.89±0.22*	1.3±0.52	-0.34 (-0.59;-0.1)	*<*0.05
Glucose (mg/dL)	90.6±7.3	95.1±11.0	-4.5 (-10.51;1.43)	0.13
Insulin (µIU/mL)	8.5±3.4	15.5±15.6	-7.0 (-14.21; 0.29)	0.059
HOMA-IR	1.9±0.8	3.7±4.1	-1.8 (-3.72; 0.09)	0.06
